# Predictors of fitness to practise declarations in UK medical undergraduates

**DOI:** 10.1186/s12909-018-1167-5

**Published:** 2018-04-05

**Authors:** Lewis W. Paton, Paul A. Tiffin, Daniel Smith, Jon S. Dowell, Lazaro M. Mwandigha

**Affiliations:** 1 Department of Health Sciences, University of York, Heslington, York, YO10 5DD UK; 2 General Medical Council, Regent’s Place, London, NW1 3JN UK; 3 School of Medicine Deanery, University of Dundee, Dundee, DD1 9SY UK

**Keywords:** Fitness to practise, UK medical undergraduates, Professionalism

## Abstract

**Background:**

Misconduct during medical school predicts subsequent fitness to practise (FtP) events in doctors, but relatively little is known about which factors are associated with such issues during undergraduate education. This study exploits the newly created UK medical education database (UKMED), with the aim of identifying predictors of conduct or health-related issues that could potentially impair FtP. The findings would have implications for policies related to both the selection and support of medical students.

**Methods:**

Data were available for 14,379 students obtaining provisional registration with the General Medical Council who started medical school in 2007 and 2008. FtP declarations made by students were available, as were various educational and demographic predictor variables, including self-report ‘personality measures’ for students who participated in UK Clinical Aptitude Test (UKCAT) pilot studies. Univariable and multivariable logistic regression models were developed to evaluate the predictors of FtP declarations.

**Results:**

Significant univariable predictors (*p* < 0.05) for conduct-related declarations included male gender, white ethnicity and a non-professional parental background. Male gender (OR 3.07) and higher ‘self-esteem’ (OR 1.45) were independently associated with an increased risk of a conduct issue.

Female gender, a non-professional background, and lower self-reported ‘confidence’ were, among others, associated with increased odds of a health-related declaration. Only ‘confidence’ was a significant independent predictor of a health declaration (OR 0.69). Female gender, higher UKCAT score, a non-professional background and lower ‘confidence’ scores were significant predictors of reported depression, and the latter two variables were independent predictors of declared depression.

**Conclusions:**

White ethnicity and UK nationality were associated with increased odds of both conduct and health-related declarations, as were certain personality traits. Students from non-professional backgrounds may be at increased risk of depression and therefore could benefit from targeted support. The small effect sizes observed for the ‘personality measures’ suggest they would offer little potential benefit for selection, over and above those measures already in use.

**Electronic supplementary material:**

The online version of this article (10.1186/s12909-018-1167-5) contains supplementary material, which is available to authorized users.

## Background

Alongside clinical competency, doctors are also expected to exhibit professional behaviour [1]. In order to practise medicine in the UK the General Medical Council (GMC), the medical regulator, requires medical students to obtain provisional registration prior to undertaking their first year as doctors (‘Foundation Year 1’). A doctor can then apply for full registration following successful completion of this first year in clinical practise. As part of the provisional registration process, students must report any significant behavioural and health concerns which may impact on their fitness to practise (FtP) [2]. In 2014, out of 7464 provisional registrations, 744 included one or more self-reported declarations. In 2015, 884 applications out of 7522 included at least one declaration. Occasionally the issue declared leads to an application for provisional registration being delayed or declined. This occurred in only four instances in 2014 and six in 2015 [3, 4].

Previous research suggests that those with conduct issues occurring during undergraduate training may be ‘at risk’ of future censure regarding breaches of professionalism. This is largely based on the findings from North American case-control studies [5–7] where medical school concerns were associated with the risk of future professional misconduct. A follow-up study supported these findings [8], although these conclusions are not universally accepted [9–11]. The feasibility of any such screening out approach has been questioned, arguing that even a good test will result in many false positives when prevalence is low.

Competition for places to study medicine is high; in the UK there are around 11 applications for every place [12]. Traditionally, selection to medical education has primarily been based on academic performance in secondary (high) school examinations. However, the competition for places, as well as a desire to widen access to the profession, led to the development of aptitude tests as part of the selection process. In the UK, this led to the introduction of the UK Clinical Aptitude Test (UKCAT) in 2006, which at the time consisted of four cognitive scales (‘abstract reasoning’, ‘decision making’, ‘quantitative reasoning’, and ‘verbal reasoning’*)*. Both secondary school grades and cognitive performance on such aptitude tests have been shown to predict subsequent academic performance in medical school [13–15]. Moreover, lower scores on the Medical College Admissions Test (MCAT) and poorer academic performance during undergraduate training (which is also associated with prior educational attainment) may be associated with later malpractice events [5].

More recently, there has been an interest in evaluating whether non-academic attributes will predict subsequent aspects of job performance once doctors are in practise. To this end, a number of self-report personal qualities questionnaires were piloted as part of the UKCAT administration, in an attempt to measure personality traits (or characteristics which could be termed ‘non-cognitive’) of those who apply to medical school. Although they were never used in the selection process, longitudinal studies at one UK medical school reported that these non-cognitive tests were predictors of both academic performance and professional behaviour in that medical school [16, 17]. However, a recent study [18] reported that the scores from these scales generally had little or no relationship with exit performance at medical school, although the authors’ noted that self-reported ‘aloofness’ and ‘empathy’ scores were independent predictors of the Situational Judgment Test (SJT) scores used for selection into the foundation programme [19]. Furthermore, ‘emotional non-defensiveness’ scores were independent predictors of both SJT score and the Educational Performance Measure (EPM), a measure of both clinical and non-clinical skills [20]. What is currently unknown is how the scores from both the cognitive and non-academic measures may relate to mental health issues in medical undergraduates.

The recent creation of a large scale repository of longitudinal data relating to medical education permits the linkage of information relating to admissions processes to later outcomes. The UK Medical Education Database (UKMED) contains a wide range of pre-admissions metrics with outcome markers such as progression through medical school and provisional registration data [21, 22]. The UKMED was formed as part of a national initiative and collaboration between a variety of UK medical education stakeholders including the GMC, Medical Schools Council and the UKCAT consortium. Thus the UKMED offers an opportunity to model the relationship between a variety of predictors at selection and later FtP concerns in medical school. To our knowledge, the present study is one of the first studies to utilise the UKMED system.

The aims of this study were thus;

i) To evaluate the feasibility of analysing the relevant data using the UKMED system
ii) To identify the educational and demographic predictors of both health and conduct-related declarations at provisional registration in UK medical students
iii) To identify any non-academic attributes that are associated with these outcomes

Depending on the strength of any associations, the findings could have implications for selection policy. In addition, the identification of sub-groups of students at elevated risk of either conduct or health issues would lead to considering the possibility of targeted support strategies.

## Methods

### Data availability and preparation

The UKMED system involves all relevant data being placed in a ‘safe haven’ where analysis occurs within the secure environment. Only summary reports and results, not individual data, are permitted to be extracted. This prevents potentially identifiable data leakage. Access to the data is obtained via an application to the UKMED research subgroup that must approve each project proposal [23].

Data were available from the UKMED in de-identified form for 14, 379 students who started medical school in 2007 and 2008 and had self-reported declarations (including those students who reported no FtP concerns). It is recognised that these data are highly sensitive and that great care was required to ensure individuals could not be identified. Therefore, the data were blunted by the GMC so that the most severe offences were categorised broadly enough to avoid identifying individual students.

### Outcome variables

The dependant variables of interest are the declarations made by the students to the GMC at provisional registration. These outcomes were derived in two ways from the data within the UKMED.

Firstly, at provisional registration students were presented with 13 pre-determined categories of declaration and selected which, if any, represented any issues that they had experienced at any time in their life. Thus, some caution must be exercised when considering the effect of age, as older students will naturally have had more time in which to experience any issues. These categories included both health issues which could impact on fitness to practise and conduct issues, such as receiving a criminal conviction. The full list is shown in Table 1. Students could select more than one category if necessary. In addition to the 13 pre-determined categories, we grouped the declarations into two further categories. Specifically, we determined whether a student had any declaration (that is, whether a student had selected one or more of the 13 declaration categories), and whether a student had any conduct-related declarations (i.e. those related to professionalism issues or legal infringements). Declarations are only valid for 3 months after they are made. Thus, a small number of individuals had to re-declare any relevant issues, for example if they deferred registration for a year. These re-declarations were few in number, and, since they were simply re-declaring the same information, all individuals with a second declaration had an equivalent original declaration present. Hence only the initial declarations were used in the analyses.

**Table 1:** Numbers of students with declarations in each category

Declaration	Outcome 1	Outcome 2
Any FtP declaration	1205	22
Conduct-related declarations		
Cautions/convictions	255	7
Other conduct issues	28	2
Current or future proceedings	10	1
Disciplinary action by employer	21	1
Fined or warned by regulator	14	0
Fixed penalty notice	321	2
Formal disciplinary action taken by medical school or university	270	11
Penalty notice for disorder or harassment	55	2
Potentially refused CGS by MRA in countries worked as a doctor	2	0
Refused registration or licence to practise	6	5
Settlement regarding malpractice or negligence	2	0
Suspended from duty, or a complaint upheld	7	0
*Any conduct-related declaration*	*777*	*18*
Health-related declarations		
Health issues that may potentially affect fitness	427	7

Secondly, in addition to selection from a list of categories, students could provide free text descriptions about any FtP issues. From these text descriptions 42 more specific categories of event were derived. These were generated by one of the authors (DS). The full list of these ‘coded outcomes’ is available in Additional file 1. There may have been a subjective element to the categorisation of the free text declarations. Thus, agreement was evaluated by providing a random sample of 98 declarations to be categorised by three of the authors (PAT, DS and JSD). Inter-rater reliability was evaluated and indexed via a Fleiss kappa value [24].

In this paper we focus on three particular outcome variables: declarations relating to conduct issues; declarations relating to health issues, and specifically; students reporting previous episodes of depression. We particularly highlight the latter outcome as depression was one of the most common categories of health declaration. Additionally there are implications for identification and support of affected students. Furthermore, depression in medical school students may be a common occurrence. For example, a study in the USA reported that approximately 15% of medical students had experienced depression [25]. Results relating to other outcome variables are included in Additional file 1.

### Predictor variables

The UKMED holds sociodemographic and educational data on entrants to UK medical schools. Most British medical schools use the UKCAT as part of their selection process. Since applicants can sit the UKCAT once in each application cycle, some entrants had multiple UKCAT scores. For these entrants the score at the most recent sitting was used, since it was this score on which their selection was based on. At the time of the study the UKCAT consisted of four cognitive scales (abstract reasoning, decision analysis, quantitative reasoning and verbal reasoning). The scale scores and the overall (total) score were standardised as z-scores within each cohort of test-takers and were used as independent variables in our models.

In addition to the four cognitive scales, between 2007 and 2010 the UKCAT trialled tests which aimed to measure ‘non-cognitive’ abilities i.e. scales intended to measure personal attributes. These tests were self-report questionnaires that asked a respondent to report on non-academic, personal qualities. We use the term ‘non-cognitive’ as shorthand for these tests, accepting that such traits are likely to have cognitive element (e.g. social cognition). The results of these assessments were not communicated to the universities and were not used as part of the admissions process. This was made clear to the candidates at the time of administration. UKCAT candidates were randomly allocated to one of four tests [26]:

- The Interpersonal Traits Questionnaire (ITQ100). This consists of four domains: ‘aloofness’ (A), ‘confidence’ (C), ‘empathy’ (E) and ‘narcissism’ (N). Scores on these domains are combined to create the ‘NACE’ measure, using the calculation C + E – (N + A) [27, 28].
- The Interpersonal Values Questionnaire (IVQ49). This has a single domain, the ‘libertarian-communitarian’ measure [28, 29]. This concept refers to the degree to which a respondent places importance on individualism as opposed to a community or societal perspective on life.
- A combination of the above two tests, the IVQ33/ITQ50, which contains the five domains present in the ITQ100 and IVQ49.
- The Managing Emotions and Resilience Scales (MEARS). This test is comprised of five domains: ‘control’, ‘faking’, ‘emotional non-defensiveness’, ‘self-discipline’ and ‘self-esteem’ [18].

Of the 6919 entrants who took the UKCAT in 2007, only two students had missing data for the non-cognitive tests. In total, the non-cognitive tests comprised 16 individual subscale scores, along with the NACE score for those students who took the ITQ100 or IVQ33/ITQ50. These 18 scales were converted to z-scores (within each cohort of entrants) and used as predictor variables. On the rare occasions when an entrant had taken the same non-cognitive scale twice (say, due to being selected at random for the same questionnaire at a resitting of the UKCAT) we used the scores from the first sitting.

Also available within the UKMED are the equated scores on the UK Foundation Programme (UKFP) SJT [19]. Candidates applying for entry to the foundation training programme are presented with a variety of written scenarios that challenge professionalism and must rank a list of possible actions in order of appropriateness. The UKFP SJT can thus be thought of as a measure of knowledge of professional behaviour. Therefore the relationship between performance on the UKFP SJT and conduct-related FtP declarations is of interest. Additionally, unlike other post-entry examinations we had access to, the UKFP SJT provided a national measure of performance. The resulting scores were standardised as z-scores within cohorts.

Data were also available for a student’s score on the deciles component of the Educational Performance Measure (EPM) [20]. The EPM provides a summary measure of a student’s clinical and non-clinical skills during medical school in comparison to their peers within their year of their medical school. An alternative version of the EPM is also available which includes rankings based on additional points for an additional degree and up to two scientific publications. However, this form was not used for the present study as it may skew the measure in favour of students who undertook an additional degree as part of their undergraduate studies. We dichotomised the EPM into two categories- those students in the top 50% of their year at their university and those in the lower 50%. The reason for this choice of classification is because, for those students who graduated in 2013, the scoring system was changed from a quartile rating to a decile rating. Thus, only a two-quantile categorisation allowed for cross comparison between these two rating systems.

Prior academic attainment was used for those students who sat A-levels. The Universities and Colleges Admissions Service (UCAS) allocate each grade at A-level a set number of UCAS points, at the time ranging from 40 points for an E-grade to 120 points for an A-grade (the data predates the introduction of A* grades). Similarly to previous research [30], we used the score for an entrant’s best three A-level results (therefore a maximum tariff of 360 points) excluding resits and performance on ‘general studies’, ‘critical thinking’, and ‘thinking skills’.

In line with previous research [13], self-reported ethnicity was dichotomised into white and non-white. School type was dichotomised into selective (including state-funded selective schools, such as ‘grammar schools’) and non-selective schools. Year of birth was available and used to dichotomise entrants into those who were 20 or older at their most recent UKCAT sitting (i.e. in order to identify ‘mature applicants’). The UKMED, via the Higher Education Statistics Agency (HESA) [31] records reported socioeconomic status, using the 8-point National Office of Statistics NS-SEC analytic classes [32]. Those who rated their parents (or their own, if they were 21 years of age or older at the time of their application to medical school) socioeconomic status as 6, 7 or 8 were classified as being from a ‘non-professional’ background. Residency was dichotomised into those living in the UK at the time of application, and those residing outside of the country. A summary of the sociodemographic data, along with completeness information, is shown in Table 2.

**Table 2:** Completeness of demographic data in the UKMED for those students with self-reported declarations

Variable	Proportion (%)	Missing (%)
Male gender	6227/14,379 (43.3)	0/14,379 (0)
Non-selective secondary school attended	9280/13,120 (70.7)	1260/14,380 (8.8)
Non-white ethnicity	4531/14,308 (31.7)	71/14,379 (0.50)
UK resident	13,080/14,380 (91.0)	0/14,380 (0)
Non-professional background	1310/11,385 (11.5)	2995/14,380 (20.8)
Age ≥ 20 at UKCAT sitting	4099/14,379 (28.5)	0/14,379 (0)

Note that some data has been rounded to the nearest multiple of five in order to comply with HESA statistical disclosure controls [58]

### Data analysis

Not all medical school students in the UKMED participated in the non-cognitive trials. Thus in order to assess the extent that the demographics differed between those entrants who took the non-cognitive tests and those entrants who did not, Pearson chi-squared tests were performed. Similarly, among those individuals who did take part in the non-cognitive trials, we also tested whether the demographics differed across each of the four non-cognitive tests. Furthermore, we also tested for differences in the distributions of scores between all UKCAT candidates in 2007 (using data obtained from a UKCAT internal report [33]) and those who eventually entered a UKCAT consortium medical school.

We also evaluated the effect of the specific medical school attended on the overall likelihood of making a declaration via a variance components model to assess the effects of university on the rate of both health and conduct-related declarations. Although students were required to self-report FtP concerns to the GMC and these were not specifically corroborated with the medical schools, there was opportunity for the forms to be reviewed before submission. Thus variation might have been introduced by differences in both culture and reporting practice between medical schools.

For each outcome, univariable logistic regression analysis was performed for each predictor variable in order to evaluate the unadjusted relationships between each of the predictor variables and each of the FtP outcomes. Informed by these results, multivariable logistic regression models were built to evaluate the adjusted relationships. Starting from a model which contained all demographic variables, standardised SJT score, dichotomised EPM performance, standardised UKCAT scores, and any significant non-cognitive relationships, stepwise backwards elimination was performed at the *p* = 0.05 significance level. At each step, a non-significant variable was eliminated from the model, until only significant variables remained at the *p* = 0.05 level. The remaining model is our final adjusted model for a particular FtP outcome. In order to avoid co-linearity, only subscales from any one non-cognitive test were included in any one multivariable model. Therefore, some outcomes have multiple multivariable models. Additionally, only observations with complete data for all of the included variables were included in each multivariable model built. This was to achieve ‘true nesting’ of the models at each step, so that the likelihood ratio tests to compare successive models were valid.

Previous work [13] has explored the potential for the UKCAT to be used as a screening tool to identify students with a high risk of failing examinations in medical school, using Receiver Operator Characteristic (ROC) curve analysis. We used the same methodology to conceptualise the (standardised) subscale scores of the non-cognitive tests as screening tools for conduct-related FtP declarations. Our choice of which non-cognitive scale scores to use for these analyses was informed by the univariable logistic regression analyses.

All data analyses were conducted using STATA MP Version 14 [34].

## Results

**Fig. 1:**
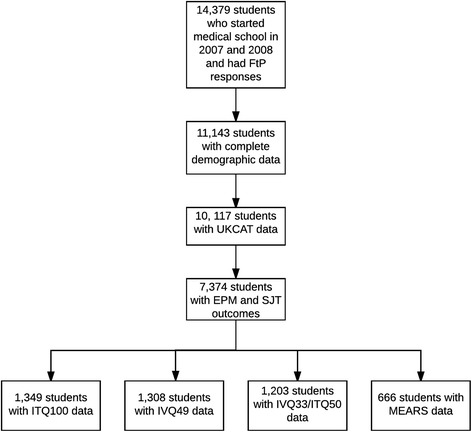
A chart showing the flow of data through the study

Figure 1 depicts the flow of data in the study. Fleiss kappa for the agreement of categorising the free text outcomes into coded outcomes was 0.65, indicating reasonable agreement. The Pearson chi-squared tests showed that those from non-professional backgrounds (*p* < 0.001), non-selective schools (*p* = 0.03) and UK citizens (*p* < 0.001) were more likely to have taken the UKCAT non-cognitive tests, whilst older entrants (*p* < 0.001) were less likely to have taken the tests. Gender (*p* = 0.79) and ethnicity (*p* = 0.80) were not significantly different between the two populations.

For those that participated in the non-cognitive trials, Pearson chi-squared tests indicated that older entrants were more likely to have been allocated to the MEARS test than the other three non-cognitive tests, and non-white students were more likely to have been randomly allocated to the IVQ33/ITQ50 than another non-cognitive scale. There were no differences in gender (*p* = 0.62), socioeconomic background (*p* = 0.39), school type attended (*p* = 0.59), and UK residential status (*p* = 0.33) across the four non-cognitive test populations.

For those who sat the UKCAT in 2007, there was evidence of range restriction between applicants and entrants in most cases (at the *p* = 0.05 level). Only the ‘libertarian-communitarian’ scale showed no evidence of range restriction.

The intraclass correlation for medical schools was very small (< 0.03 for conduct-related declarations and < 0.02 for health declarations) suggesting only trivial overall effects of university on declarations.

### Conduct-related outcomes

#### Univariable analysis

**Fig. 2:**
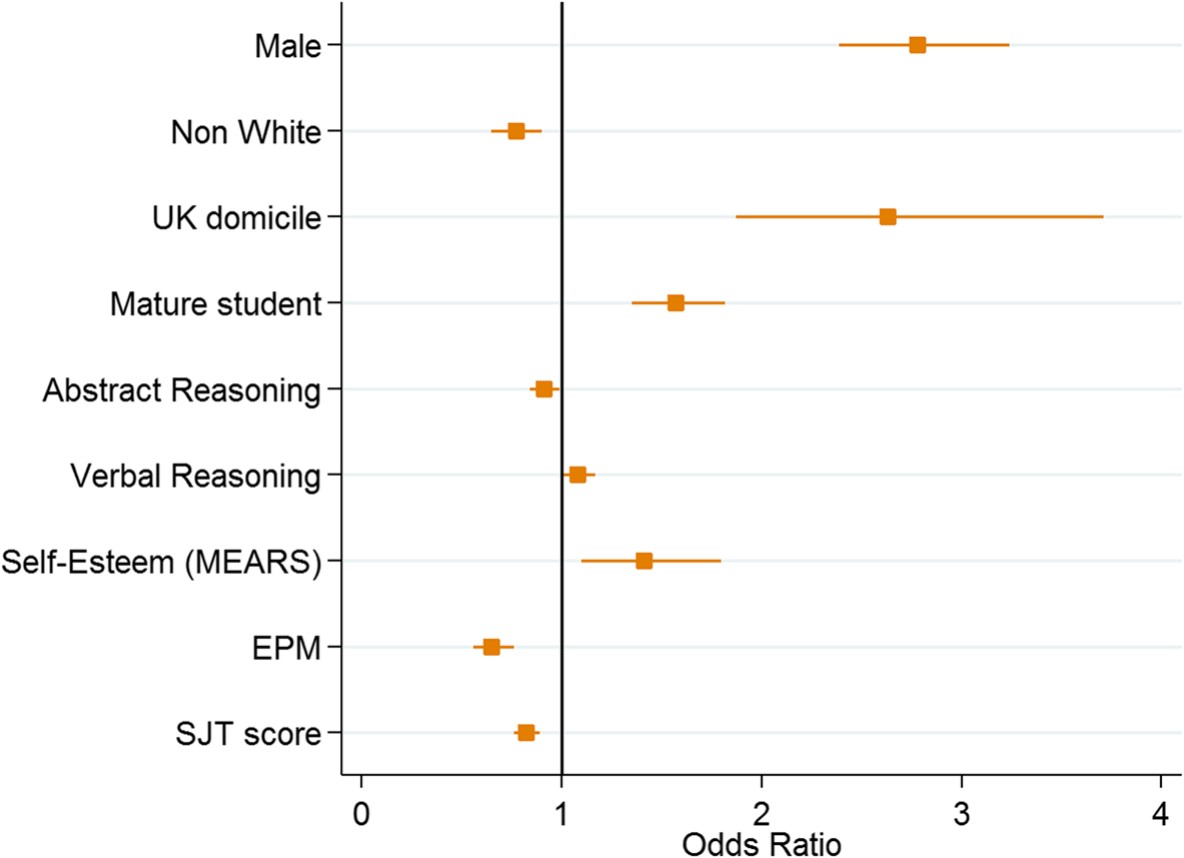
Significant odds ratios and confidence intervals from the univariable analyses predicting conduct-related declarations

Figure 2 depicts the odds ratios and associated confidence intervals for the statistically significant predictors from the univariable analyses for conduct-related declarations. We can see that the odds of a male student having an FtP declaration were 2.78 times higher than female students. Students living in the UK (OR 2.63), mature students (OR 1.57), higher (standardised) verbal reasoning scores (OR 1.08), and higher (standardised) scores on the ‘self-esteem’ component of the MEARS (OR 1.40) had higher odds of a conduct-related declaration. In the latter case this observation can be interpreted as follows; for every standard deviation above the mean scored on the self-esteem scale, an entrant had 40% higher odds of declaring a conduct-related issue.

EPM ranking (OR 0.65), performance on the foundation programme SJT (OR 0.82), higher scores in abstract reasoning (OR 0.91), and being non-white (OR 0.77) were all associated with lower odds of declaring a conduct-related issue.

#### Multivariable analysis

The full multivariable model for the conduct-related declarations included the ‘self-esteem’ score on the MEARS. The stepwise regression procedure returns a model with only male gender (OR 3.07) and ‘self-esteem’ score (OR 1.45) as independent variables (see Table 3).

**Table 3:** Results from the backwards stepwise multivariable logistic regression model, where *MEARS: self-esteem* is included in the initial model

Predictor variable	Odds Ratio	*p*-value	95% Confidence Interval
Conduct-related declaration (*N* = 770)			
Male gender	3.07	0.011	(1.29, 7.32)
MEARS: self-esteem	1.45	0.039	(1.02, 2.06)

### Declared health issues

#### Univariable analysis

**Fig. 3:**
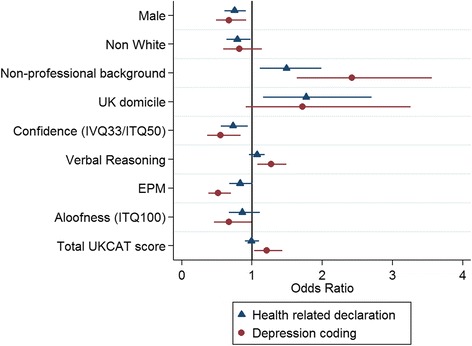
Odds ratios and confidence intervals for the univariable analyses for health related declarations and the coded depression outcome. Only outcomes with at least one significant predictor are included

Figure 3 shows the univariable analyses for declared health issues as the outcome (blue triangle) and also for analyses relating to the free-text coded outcome ‘depression’ (red circle). Predictors with statistically significant relationships (*p* < 0.05) with at least one of these two outcomes are included in Fig. 3. Any 95% confidence interval which includes 1 is not statistically significant.

Males were less likely to declare an issue relating to health than females (OR 0.75). Those students from non-professional backgrounds were more likely to declare health issues (OR 1.49), as were students living in the UK before medical school (OR 1.77). Furthermore more confident students, as reported via the IVQ33/ITQ50 test scores, had lower odds of declaring a health issue (OR 0.73).

Males had lower odds of declaring depression compared to females (OR 0.67). Students from a non-professional background had over twice the odds of declaring depression compared to those from a professional background (OR 2.42). Higher UKCAT score (OR 1.21) and better performance on the verbal reasoning component of the UKCAT (OR 1.27) were both associated with increased odds of declaring depression. Higher scores on the ‘confidence’ subscale of the IVQ33/ITQ50 were significantly associated with lower odds of declaring depression (OR 0.55).

#### Multivariable analysis

The results for the multivariable analyses for health-related outcomes are presented in Table 4. The multivariable model for health declarations contains only one independent variable: ‘confidence’ score on the IVQ33/ITQ50 test. Higher scores on this scale are associated with reduced odds of declaring a health issue (OR 0.69).

**Table 4:** Results from three separate backwards stepwise multivariable models, relating to health related declarations

Predictor variable	Odds Ratio	*p*-value	95% Confidence Interval
Health declaration (*N* = 1459)			
IVQ33/ITQ50: confidence	0.69	0.017	(0.51, 0.94)
Depression coding: IVQ33ITQ50 model (*N* = 1459)			
Non-professional background	4.39	0.010	(1.44, 13.40)
IVQ33/ITQ50: confidence	0.52	0.013	(0.31, 0.87)
Depression coding: ITQ100 model (*N* = 1619)			
Non-professional background	4.78	0.001	(1.97, 11.60)

The univariable analyses found that ‘aloofness’, as measured by the ITQ100, and ‘confidence’, as measured by the IVQ33/ITQ50, were both significantly associated with reduced odds of reporting the coded outcome ‘depression’. Therefore, two multivariable models were built, one for each of these non-cognitive predictors. Thus, these two models were built on two distinct data sets. Table 4 shows that both of the multivariable models for depression report that a non-professional background is a significant independent predictor of declaring depression, and in both models the odds ratio is greater than four.

### The potential of the non-cognitive tests to screen for a high risk of conduct problems

**Fig. 4:**
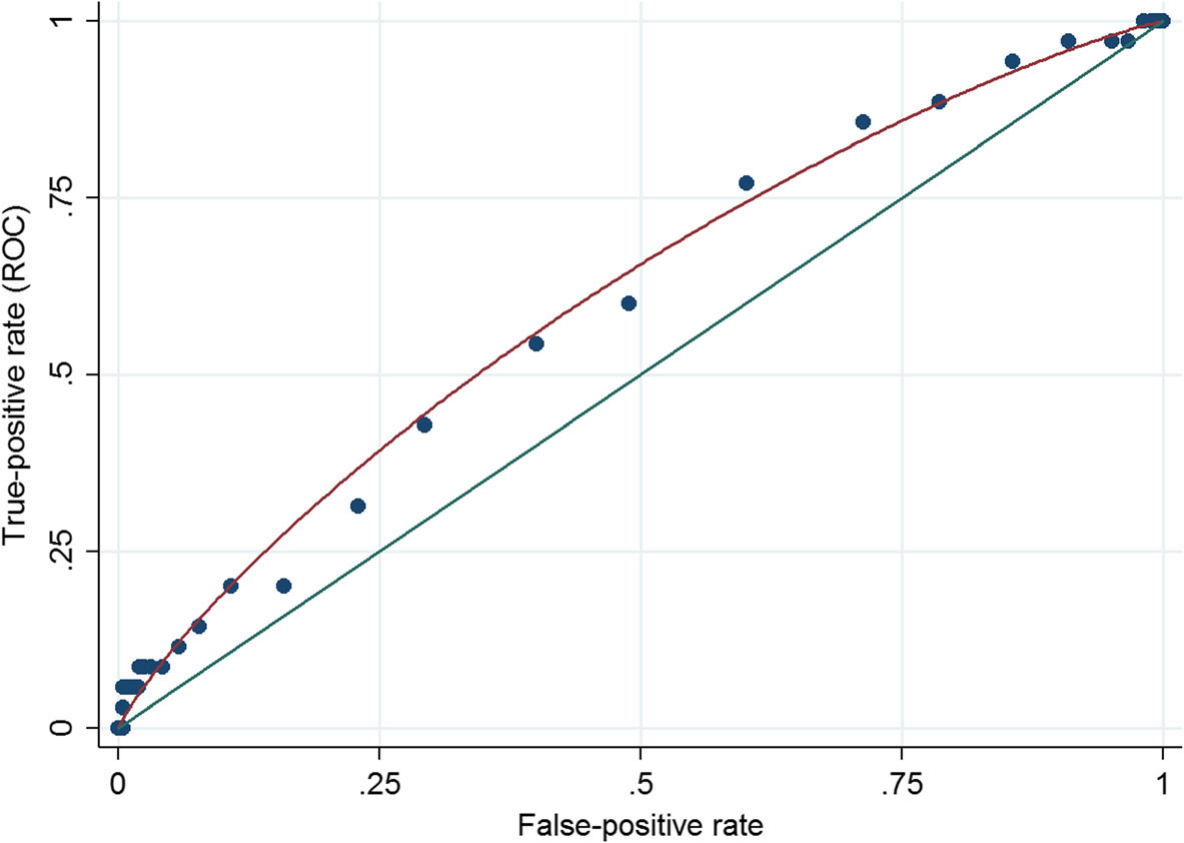
ROC curve for the use of the MEARS ‘self-esteem’ score as a tool to screen out students who are likely to have at least one conduct-related FtP declaration

As can be seen in Fig. 2, there is a strong univariable relationship between ‘self-esteem’ (as measured by the MEARS test) and declaring at least one conduct-related FtP issue. Using an ROC curve, we can conceptualise this test as a screening tool for conduct-related declarations. Figure 4 shows a reasonably flat ROC curve, although the area under the curve (AUC) is 0.61 (95% confidence interval for AUC 0.52 to 0.71). For illustrative purposes we can hypothesise the potential screening threshold as the mean score on the ‘self-esteem’ scale of the MEARS (that is, a standardised z-score of zero) [13]. We can construct a two-by-two contingency table for this cut off score (Table 5). This table shows that 4.4% (21/480) of those who screen positive (that is, have above average MEARS ‘self-esteem’ score) have at least one conduct-related declaration. In contrast, only 2.8% (14/494) who screened negative have at least one such declaration. That is, the positive predictive value of the ‘self-esteem’ scale of the MEARS is 4.4%, and the absolute risk reduction from such a hypothetical screening process is 1.6%. The ‘number needed to reject’ [13] is approximately 22 (resulting from the ratio 459:21). That is, if the MEARS was used as a screening tool to identify entrants who may report conduct-related FtP issues, 22 students who do not report such incidents would need to be ‘rejected’ in order to screen out one student with at least one conduct-related declaration.

**Table 5:** Two-by-two contingency table for the ‘self-esteem’ score on the MEARS test as a hypothetical screening test for conduct-related declarations

	At least one conduct-related declaration?		
Above average self-esteem*?*	No	Yes	Total
No	480	14	494
Yes	459	21	480
Total	939	35	974

## Discussion

To our knowledge, this study is one of the first to demonstrate the feasibility of analysing national data relating to medical education from within the safe haven environment of the UKMED.

### Conduct-related declarations

We observed a number of univariable associations between the predictors and the odds of a conduct-related event being declared. In line with previous findings on FtP in qualified doctors we noted that male gender was a risk factor for conduct concerns [35, 36]. Additionally, we found that UK domiciles and ‘mature’ students had increased odds of a conduct-related issue being declared. In contrast, reported non-white ethnicity was associated with a reduced odds ratio of such a declaration. Furthermore, superior SJT performance and higher EPM performance were both associated with reduced odds of a conduct-related declaration. The scales of the UKCAT had some ability to predict conduct-related declarations: higher scores in verbal reasoning were associated with increased odds of a recorded conduct-related declaration, whereas higher scores in abstract reasoning were associated with reduced odds. Only one non-cognitive scale was a significant predictor of conduct-related declarations: self-reported ‘self-esteem’ on the MEARS test*.*

In terms of independent risk factors, only male gender and ‘self-esteem*’* remain significant predictors of conduct-related declarations (among those who took the MEARS test).

### Health related declarations

Many of the univariable relationships for health declarations remain the same as for conduct-related declarations. For example, in both cases, white ethnicity and residence in the UK had increased odds of a declaration. However, a key difference was that female gender was associated with increased odds of health concerns being reported.

Overall, the results relating to depression display similar patterns to those for health declarations in general. This is unsurprising given that depression accounted for almost half of all health-related declarations. Increased performance on the verbal reasoning scale of the UKCAT, as well as total UKCAT score, were associated with increased odds of depression. We observed that higher ‘confidence’ scores on the IVQ33/ITQ50 were associated with reduced odds of both depression and health declarations in general.

In contrast to the conduct-related FtP declarations, gender was not independently associated with the odds of a health declaration. Self-reported ‘confidence’ prior to entering university, as measured by the IVQ33/ITQ50, is the only factor independently associated with the risk of a health declaration being reported. According to our multivariable model, for those students who took the IVQ33/ITQ50 non-cognitive test, only ‘confidence’ scores and being from a non-professional background were statistically significant predictors of depression being reported. For those students who took the ITQ100, only non-professional background remained independently associated with the risk of depression being reported. In both models, being from a non-professional background was a very strong predictor of declaring depression during undergraduate medical education, a result consistent with previous findings [37].

#### Possible interpretations

There is some evidence from our analyses that those who self-report higher self-esteem are more likely to experience conduct-related issues during their time at medical school. A conduct-related FtP issue could be considered as a behavioural issue, and it is possible that the MEARS ‘self-esteem’ score may actually be reflecting a certain amount of aloofness or narcissism, rather than a healthy sense of self-worth as such. This is also supported by the observation that the MEARS ‘self-esteem’ score was not predictive of EPM [18]. Thus, it may be leading to an increased risk of a behavioural issue and therefore conduct-related FtP declarations.

We observed a significant univariable relationship between increased performance on the UK Foundation Programme SJT and reduced odds of declaring a conduct-related issue. As the SJT can be thought of as measuring a candidate’s knowledge of professional behaviour, our results suggest there is a link between knowledge of professional behaviour and exhibiting professional behaviour. This provides some circumstantial evidence to support the use of SJTs during the undergraduate selection process. This assumes that SJTs used to select into medical school measure similar constructs to those administered at later career stages. Certainly an SJT has already been introduced as a component of the UKCAT [38], and there are plans to extend such testing in other countries.

Students who most recently sat the UKCAT at the age of 20 or older, and therefore are classed as ‘mature’ students, had increased univariable odds of a conduct-related issue being declared. As declarations are based on events at any point in life, this result may simply be an artefact of older students extra years of life. From Table 1 we can see that a significant proportion of conduct related FtP declarations were fixed penalty notices, of which many would be traffic violations. We assume older students are more likely to have traffic offences simply because they will have spent longer driving than younger students. That being said, there is also the possibility that ‘mature’ students, perhaps somewhat counterintuitively, are in fact more likely to experience conduct-related issues during medical school. If this is the case, the reasons for this would be worthy of further investigation.

When considering the univariable relationship between conduct-related FtP declarations and the cognitive scales of the UKCAT, given that gender is a strong predictor, the findings should be interpreted in the light of any known gender differences observed for the subtest scores. The puzzling association between higher verbal reasoning scores and conduct-related declarations could be explained by the fact that males tend to outperform females on this aspect of the test [39]. Thus, the scores may be a proxy marker for male gender. In contrast, abstract reasoning scores were somewhat protective against conduct-related declarations, and this UKCAT scale does not seem to exhibit any significant gender bias [39]. Those who are better able to logically reason situations are perhaps better suited at avoiding situations which could lead to a behavioural issue.

There are a number of univariable relationships between health issues and sociodemographic variables present. Female gender is a significant univariable predictor of declaring a health issue at provisional registration. This may simply be an artefact of females being more likely to seek medical advice than males [40]. However, when considering independent predictors, only ‘self-confidence’ score on the IVQ33/ITQ50 remains as a significant predictor. It is possible that those students who have higher self-confidence on entrance to medical school can cope better with the pressures of medical school, and thus are less likely to suffer from health issues, in particular mental health difficulties. This interpretation is supported by the results for depression, where the ‘self-confidence’ score, again as measured by the IVQ33/ITQ50*,* is both a univariable and multivariable predictor (for those who completed this particular scale) of such an outcome. It has previously been reported that female medical students are less confident than their male counterparts [41]. It is therefore possible that self-confidence is acting as a proxy variable for gender. If this was the case the known gender differences with regards to seeking medical advice [40] may play a role, even though gender is not an independent predictor of declaring a health related issue at provisional registration.

Being from a self-reported non-professional background appears to be a significant independent predictor of reporting depression during undergraduate medical training. Depression rates in medical school students have been the subject of previous research. A meta-analysis of 77 studies of depression in students studying medicine estimated the global prevalence to be 28.0% [42]. The same meta-analysis also reported no significant difference between depression rates in medical students and those studying other subjects from six relevant studies [42]. However, an American study reported that medical students are more likely to suffer from depression than their peers not attending university [43].

While factors such as perceived stress are associated with the risk of depression in the student population [44], this study, and others, has observed that students from non-professional backgrounds had an additional risk of reporting depression compared to those from professional backgrounds. A European-wide study [45] has shown a significant association between lower socioeconomic status and higher prevalence of depression. It is well established that students from non-professional backgrounds are under-represented in medical schools in the UK [30, 46]. Interestingly an American multi-school study found that African-American students, an under-represented group in the US medical education system [47], were more likely to experience episodes of depression during their medical training [48]. It has also been reported that anxiety in Brazilian medical students is linked to being on a tuition scholarship [49]. It is possible that being from an under-represented group at medical school brings additional challenges, thus increasing the risk of depression in that subpopulation of students.

#### Potential strengths and limitations

An obvious strength of this study was the sample size, consisting of two full cohorts of UK medical graduates, based on access to the UK Medical Education Database. The main limitation of this work is outcome variable itself. Fitness to Practise declarations were self-reported and so may underestimate the true population values, especially if they were perceived to be minor or not directly related with medical practise (such as fixed penalty notices for speeding). In the future, cross validation with medical school records would be important to ensure the accuracy of declarations. Moreover, the relationships between the FtP declarations and the independent variables are complex. For example, it may be that non-white students may be less likely to report mental health problems due to the cultural variation in perceived stigma [50].

Secondly, defining the category of declarations from the coded outcomes was subjective to some extent, although good rater agreement was shown on analysis. It should be borne in mind that the personal qualities instruments were piloted in low stakes conditions; faking effects may be more prominent in high-stakes testing [51]. Previous studies using the Personal Qualities Assessment have sometimes used scores from the constituent scales to classify respondents [52]. However, we did not feel the evidence supported such a categorical approach to the instrument scores. The need to dichotomise EPM will have resulted in a loss of information and power. Thus, this study may have underestimated the link between academic performance at medical school and FtP declarations.

Although some demographic data were missing, most notably for socioeconomic background where 22% of observations were absent, the dataset was relatively complete. Also, despite multiple analyses with outcomes that may be assumed to be non-independent to some extent, we have not applied any correction for significance (e.g. the Bonferroni correction). We leave it to the reader to decide whether the associations observed are relatively strong and of ‘educational’ significance.

We utilised our previous approach of conceptualising selection measures as diagnostic screening tests. In this case the relatively flat ROC curve, alongside the high NNR value, suggests that the ‘self-esteem’ score on the MEARS test would be a poor screening tool for conduct-related declarations. However, although the non-cognitive scales were not used as a selection criteria, it is still possible that some ‘indirect range restriction’ in either the outcome or predictor variables occurred (e.g. if some non-cognitive scores were related to a selection criterion such as A level or interview performance). Moreover, screening for uncommon outcomes will always be challenging. There are further limitations to this approach in this dataset; only data were available on entrants, and not the wider population of applicants. This means that plausible values for outcomes could not be imputed. Using data from the range-restricted entrants only is likely to have led to a flatter ROC curve. Thus, our estimates of screening characteristics may underestimate the true potential of these instruments.

A notable limitation of the study relates to the multivariable models and the non-cognitive tests. Each of the four non-cognitive tests was carried out on a different subpopulation of those entrants who participated in the UKCAT non-cognitive trials (which in turn is a sub-population of all entrants to medical school). This means that, for each outcome, we had to build a separate multivariable model for each non-cognitive test (for which there was a significant univariable relationship with the relevant outcome). Students who participated in the trials were randomly allocated to one of the four tests, a process which should have ensured no bias in the demographics of each of the four subpopulations of entrants. However, we did observe differences in the demographics between these four subpopulations. Furthermore, we also observed demographic differences between those who took part in the non-cognitive trials and those who did not. Therefore, the inferences from multivariable models which were built using a particular subpopulation of entrants can only be applied to those who took the relevant non-cognitive test.

#### Implications for policy

This study has identified a number of markers that may highlight students at particular risk of FtP issues. Although the ROC analysis and the poor NNR value imply that the ‘non-cognitive’ tests would be poor screening tools, they do appear to possess some predictive value of issues that may have implications for fitness to practise. Being able to identify students most at risk of behavioural or health issues would provide an opportunity to consider preventative measures for such groups. There are a number of factors, both educational and demographic, which are linked with an increased risk of declaring an experience of depression – in particular, being from a less advantaged background.

There are thus implications for ‘Widening Participation’ (WP) schemes. In the UK, a number of recommendations resulting from the ‘Selecting for Excellence’ report [12] have led to initiatives such as outreach schemes [53], which aim to increase the number of students from under-represented groups attending medical school. Our results suggest specific support may need to be put in place for WP entrants. This is consistent with international findings regarding the support of under-represented students [54]. Medical students could perhaps be encouraged to build up emotional resilience as part of their education, although there is debate as to the benefits of formal resilience training [55]. It may be that more general supportive approaches, such as mentoring or ‘buddying’ systems for students from under-represented groups, will enhance their long term well-being [56].

Additionally, this study has highlighted potential areas for improvement in the FtP system at provisional registration. In particular, being able to separate out those FtP issues that occurred before medical school from those that occurred during medical school would enable further investigation into the relationship between age and FtP declarations. Indeed, the GMC has already started to address this issue. From 2017 onward, medical schools are required to submit data on student FtP incidents that occurred during medical school. This has the potential to provide a more reliable outcome variable than the self-reported character declaration used for this study.

This study has also shown the potential of the UKMED as an important tool for research. The potential of following cohorts from the start of medical training right through into practise means the UKMED should become an increasingly important component of research relating to the UK medical workforce. Thus, it will be possible to perform further, future, research into the predictors of FtP issues in postgraduate training.

## Conclusions

This study has focussed on the predictors of fitness to practise issues declared at provisional registration. Being able to identify those students at risk of such issues, and being able to target preventive help at those students from high risk groups may enable more students to perform to their potential at medical school. Furthermore, previous work has found links between issues which occur during undergraduate education and future professional misconduct. If successful preventive measures could be introduced, future professional misconduct could potentially be reduced. Having demonstrated the feasibility of using the UKMED system there is also the promise of being able to follow these cohorts on into postgraduate training and clinical practise. Moreover UKMED has been expanded to encompass other entrants to medical school from 2007 to 2014, and will shortly include data on doctors in postgraduate training who obtained their primary medical qualification outside of the UK. It is thus hoped that the dataset will become an evolving and precious resource to support the creation of policy relating to the education, training and regulation of the UK medical workforce.

## Additional file

Additional file 1: Additional results. (DOCX 31 kb)

## Abbreviations

AUC: Area under the curve
EPM: Educational Performance Measure
FtP: Fitness to practise
GMC: General Medical Council
ITQ: Interpersonal Traits Questionnaire
IVQ: Interpersonal Values Questionnaire
MCAT: Medical College Admissions Test
MEARS: Managing Emotions and Resilience Scales
OR: Odds ratio
ROC: Receiver operator curve
SJT: Situational judgment test
UCAS: Universities and Colleges Admissions Service
UKCAT: UK Clinical Aptitude Test
UKFP: UK Foundation Programme
UKMED: UK Medical Education Database
WP: Widening participation
