# Supplementary material for: Predictors of fitness to practise declarations in UK medical undergraduates
Source: BMC Med Educ. 2018 Apr 5;18:68. doi: 10.1186/s12909-018-1167-5 (PMC5887261; doi:10.1186/s12909-018-1167-5)
Supplement: Supplementary file 1 — Additional results. (DOCX 31 kb) [file 12909_2018_1167_MOESM1_ESM.docx]

**Predictors of Fitness to Practice Declarations in UK Medical Undergraduates: Supplementary Appendix**

Lewis. W. Paton, Paul. A. Tiffin, Daniel Smith, Jon S. Dowell, and Lazaro M. Mwandigha

**A: Free coded text outcomes**

| **Outcome coded from free text** | **Outcome 1** | **Outcome 2** |
| --- | --- | --- |
| Violence | 15 | 1 |
| Criminal damage | 18 | 0 |
| Littering | 9 | 1 |
| Public disorder | 17 | 2 |
| Soliciting | 0 | 0 |
| Breach of the peace | 3 | 1 |
| Harassment (without violence) | 3 | 0 |
| Possession of class A drugs | 1 | 0 |
| Possession of class B or C drugs | 13 | 0 |
| Drunk and disorderly | 53 | 0 |
| Driving under the influence of alcohol | 30 | 2 |
| Fitness to practice issues related to alcohol but not disorderly | 43 | 1 |
| Driving without due care and attention | 27 | 0 |
| Speeding | 103 | 1 |
| Driving without insurance and/or tax | 28 | 1 |
| Parking | 34 | 1 |
| Other motoring offences | 167 | 3 |
| Shoplifting | 12 | 0 |
| Theft | 9 | 0 |
| Fare evasion | 61 | 0 |
| Other dishonesty | 1 | 0 |
| Academic misconduct | 17 | 3 |
| Other misconduct | 77 | 2 |
| Attendance | 32 | 1 |
| Plagiarism | 43 | 1 |
| Probity | 20 | 2 |
| Professional performance | 15 | 1 |
| Other student FtP | 3 | 0 |
| Physical health | 189 | 3 |
| Depression | 182 | 2 |
| Stress/anxiety | 54 | 3 |
| Suicide/self-harm | 4 | 0 |
| Eating/body image mental health | 22 | 0 |
| Personality disorders | 1 | 0 |
| Mania/bipolar | 17 | 0 |
| Psychosis/schizophrenia | 2 | 1 |
| Obsessive compulsive disorder | 14 | 2 |
| Other mental health issue | 13 | 0 |
| Alcohol or drug dependency | 4 | 0 |
| Drug toxicity | 1 | 0 |
| Dyslexia | 10 | 1 |
| Attention deficit hyperactivity disorder | 5 | 0 |

Table S1: Number of students with each of the outcomes coded from the free text declarations

**B.1: Additional results from univariable analyses: declared outcomes**

In all the tables that follow, ‘N’ represents the total number of individuals in the analysis, and ‘n’ the number of individuals who declared that outcome. Table S3 is represented as Figure 2 in the main text, and Tables S4 and S13 are represented as Figure 3.

| **Outcome: any FtP declaration (N = 14379, n=1205)** | | |
| --- | --- | --- |
| **Predictor** | **Odds ratio (95% CI)** | **p-value** |
| Male sex | 1.77 (1.58 to 2.00) | <0.001 |
| Non-white ethnicity | 0.74 (0.65 to 0.85) | <0.001 |
| UK resident | 2.50 (1.91 to 3.27) | <0.001 |
| Non-professional background | 1.28 (1.06 to 1.54) | 0.009 |
| Over 20 years of age at UKCAT sitting | 1.30 (1.15 to 1.47) | <0.001 |
| Verbal Reasoning (standardised) | 1.09 (1.02 to 1.16) | 0.01 |
| IVQ33/ITQ50: aloofness (standardised) | 1.18 (0.99 to 1.41) | 0.067 |
| MEARS: self-esteem (standardised) | 1.20 (0.97 to 1.49) | 0.089 |
| EPM | 0.75 (0.66 to 0.85) | <0.001 |
| SJT equated score (standardised) | 0.87 (0.82 to 0.93) | <0.001 |

Table S2: Results from univariable logistic regression with ‘any FtP declaration’ as the outcome

| **Outcome: any conduct-related declaration (N = 14379, n=777)** | | |
| --- | --- | --- |
| **Predictor** | **Odds ratio (95% CI)** | **p-value** |
| Male | 2.78 (2.39 to 3.24) | <0.001 |
| Non-white ethnicity | 0.77 (0.65 to 0.90) | 0.001 |
| UK | 2.63 (1.87 to 3.71) | <0.001 |
| Over 20 years of age at UKCAT sitting | 1.57 (1.35 to 1.82) | <0.001 |
| Abstract Reasoning (standardised) | 0.91 (0.84 to 0.99) | 0.021 |
| Decision Analysis (standardised) | 0.93 (0.86 to >1.00) | 0.065 |
| Verbal Reasoning (standardised) | 1.08 (>1.00 to 1.17) | 0.042 |
| IVQ33/ITQ50: aloofness (standardised) | 1.23 (0.98 to 1.55) | 0.070 |
| MEARS: self-esteem (standardised) | 1.41 (1.10 to 1.80) | 0.006 |
| EPM | 0.65 (0.56 to 0.76) | <0.001 |
| SJT equated score (standardised) | 0.82 (0.76 to 0.89) | <0.001 |

Table S3: Results from univariable logistic regression with ‘conduct-related’ declarations as the outcome

| **Outcome: health-related declaration (N = 14379, n=427)** | | |
| --- | --- | --- |
| **Predictor** | **Odds ratio (95% CI)** | **p-value** |
| Male | 0.75 (0.61 to 0.91) | 0.004 |
| Non-white ethnicity | 0.79 (0.64 to 0.98) | 0.036 |
| Non-professional background | 1.49 (1.11 to 1.99) | 0.008 |
| UK | 1.77 (1.16 to 2.70) | 0.008 |
| Over 20 years of age at UKCAT sitting | 0.81 (0.65 to 1.02) | 0.069 |
| IVQ33/ITQ50: confidence (standardised) | 0.73 (0.56 to 0.94) | 0.017 |
| IVQ33/ITQ50: empathy (standardised) | 1.27 (0.97 to 1.65) | 0.082 |
| EPM | 0.83 (0.68 to 1.01) | 0.056 |

Table S4: Results from univariable logistic regression with ‘health-related’ declarations as the outcome

| **Outcome: convictions (N = 14379, n=255)** | | |
| --- | --- | --- |
| **Predictor** | **Odds ratio (95% CI)** | **p-value** |
| Male | 2.81 (2.16 to 3.67) | <0.001 |
| UK | 2.75 (1.41 to 5.36) | 0.003 |
| Over 20 years of age at UKCAT sitting | 2.12 (1.66 to 2.73) | <0.001 |
| Abstract Reasoning (standardised) | 0.87 (0.76 to >1.00) | 0.053 |
| Decision Analysis (standardised) | 0.85 (0.74 to 0.97) | 0.016 |
| MEARS: faking (standardised) | 1.48 (1.02 to 2.16) | 0.039 |
| MEARS: self-discipline (standardised) | 1.71 (1.13 to 2.58) | 0.011 |
| MEARS: self-esteem (standardised) | 1.72 (1.24 to 2.40) | 0.001 |
| Best of Three A-levels | <1.00 (0.99 to <1.00) | 0.035 |
| EPM | 0.63 (0.48 to 0.83) | <0.001 |

Table S5: Results from univariable logistic regression with ‘convictions’ declared as the outcome

| **Outcome: conduct (N = 14379, n=28)** | | |
| --- | --- | --- |
| **Predictor** | **Odds ratio (95% CI)** | **p-value** |
| Male | 3.94 (1.67 to 9.27) | 0.002 |
| Non-professional background | 2.70 (1.14 to 6.40) | 0.024 |
| ITQ100: confidence (standardised) | 2.14 (0.94 to 4.88) | 0.071 |
| IVQ49: lib-com (standardised) | 0.31 (0.08 to 1.17) | 0.084 |
| EPM | 0.51 (0.24 to 1.11) | 0.091 |

Table S6: Results from univariable logistic regression with ‘conduct’ declared as the outcome

| **Outcome: proceedings (N = 14379, n=10)** | | |
| --- | --- | --- |
| **Predictor** | **Odds ratio (95% CI)** | **p-value** |
| Male | 5.24 (1.11 to 24.69) | 0.036 |
| Non-professional background | 15.46 (3.86 to 61.88) | <0.001 |

Table S7: Results from univariable logistic regression with ‘proceedings’ declared as the outcome

| **Outcome: disciplinary action by employer (N = 14379, n=21)** | | |
| --- | --- | --- |
| **Predictor** | **Odds ratio (95% CI)** | **p-value** |
| Male | 2.62 (1.06 to 6.50) | 0.037 |
| Non-professional background | 2.76 (0.99 to 7.76) | 0.052 |
| Over 20 years of age at UKCAT sitting | 6.29 (2.44 to 16.22) | <0.001 |
| ITQ100: empathy (standardised) | 0.04 (<0.001 to 1.39) | 0.074 |
| MEARS: control (standardised) | 0.09 (0.01 to 1.47) | 0.090 |
| Best of Three A Levels | 0.99 (0.98 to <1.00) | 0.002 |

Table S8: Results from univariable logistic regression with ‘disciplinary action by employer’ declared as the outcome

| **Outcome: fined (N = 14379, n=14)** | | |
| --- | --- | --- |
| **Predictor** | **Odds ratio (95% CI)** | **p-value** |
| Non-white ethnicity | 2.88 (<1.00 to 8.30) | 0.05 |
| UK | 0.18 (0.06 to 0.53) | 0.002 |
| Decision Analysis (standardised) | 0.57 (0.32 to >1.00) | 0.050 |

Table S9: Results from univariable logistic regression with ‘fined’ declared as the outcome

| **Outcome: fixed penalty (N = 14379, n=321)** | | |
| --- | --- | --- |
| **Predictor** | **Odds ratio (95% CI)** | **p-value** |
| Male | 2.64 (2.09 to 3.33) | <0.001 |
| Non-white ethnicity | 0.61 (0.47 to 0.79) | <0.001 |
| Over 20 years of age at UKCAT sitting | 2.25 (1.80 to 2.80) | <0.001 |
| Abstract Reasoning (standardised) | 0.90 (0.80 to 1.02) | 0.09 |
| Verbal Reasoning (standardised) | 1.22 (1.07 to 1.38) | 0.002 |
| IVQ33/ITQ50: lib com (standardised) | 1.48 (0.98 to 2.24) | 0.07 |
| MEARS: self-esteem (standardised) | 1.53 (0.95 to 2.46) | 0.08 |

Table S10: Results from univariable logistic regression with a ‘fixed penalty’ declared as the outcome

| **Outcome: formal disciplinary action taken by medical school (N = 14379, n=270)** | | |
| --- | --- | --- |
| **Predictor** | **Odds ratio (95% CI)** | **p-value** |
| Male | 3.23 (2.48 to 4.21) | <0.001 |
| UK | 1.98 (1.13 to 3.47) | 0.02 |
| Abstract Reasoning (standardised) | 0.88 (0.77 to 0.99) | 0.04 |
| Decision Analysis (standardised) | 0.89 (0.78 to 1.01) | 0.07 |
| Total UKCAT score (standardised) | 0.89 (0.78 to 1.01) | 0.06 |
| IVQ33/ITQ50: aloofness (standardised) | 1.82 (1.33 to 2.47) | <0.001 |
| IVQ33/ITQ50: narcissism (standardised) | 1.43 (1.03 to 1.97) | 0.031 |
| EPM | 0.39 (0.30 to 0.51) | <0.001 |

Table S11: Results from univariable logistic regression with ‘formal disciplinary action by the university’ declared as the outcome

| **Outcome: penalty notice for disorder or harassment notice (N = 14379, n=55)** | | |
| --- | --- | --- |
| **Predictor** | **Odds ratio (95% CI)** | **p-value** |
| Male | 9.04 (4.09 to 19.99) | <0.001 |
| Abstract Reasoning (standardised) | 0.77 (0.58 to 1.02) | 0.069 |
| IVQ33/ITQ50: confidence (standardised) | 0.45 (0.18 to 1.08) | 0.073 |

Table S12: Results from univariable logistic regression with a ‘penalty notice’ declared as the outcome

**B.2: Additional results from univariable analyses: coded outcomes**

| **Variable: depression (N = 14379, n=182)** | | |
| --- | --- | --- |
| **Predictor** | **Odds ratio (95% CI)** | **p-value** |
| Male | 0.67 (0.49 to 0.92) | 0.012 |
| Non-professional background | 2.42 (1.64 to 3.56) | <0.001 |
| UK | 1.72 (0.91 to 3.26) | 0.098 |
| Verbal Reasoning (standardised) | 1.27 (1.08 to 1.49) | 0.003 |
| ITQ100: aloofness (standardised) | 0.67 (0.46 to 0.99) | 0.042 |
| IVQ33/ITQ50: confidence (standardised) | 0.55 (0.36 to 0.84) | 0.005 |
| NACE score (standardised) | 1.43 (<1.00 to 2.08) | 0.060 |
| Total UKCAT score (standardised) | 1.21 (1.03 to 1.43) | 0.018 |
| EPM | 0.52 (0.38 to 0.70) | <0.001 |

Table S13: Results from univariable logistic regression with a ‘depression’ coding as the outcome

| **Outcome: speeding (N = 14379, n=103)** | | |
| --- | --- | --- |
| **Predictor** | **Odds ratio (95% CI)** | **p-value** |
| Male | 1.63 (1.10 to 2.40) | 0.014 |
| Non-white ethnicity | 0.42 (0.25 to 0.71) | 0.001 |
| UK resident | 5.05 (1.24 to 20.48) | 0.024 |
| Over 20 years of age at UKCAT sitting | 4.53 (3.02 to 6.79) | <0.001 |
| Verbal Reasoning (standardised) | 1.27 (<1.00 to 1.61) | 0.054 |
| MEARS: control (standardised) | 2.34 (1.18 to 4.63) | 0.014 |
| MEARS: emotional non-defensiveness (standardised) | 2.04 (1.25 to 3.34) | 0.005 |
| MEARS: faking (standardised) | 2.17 (1.25 to 3.80) | 0.006 |
| MEARS: optimism (standardised) | 2.36 (1.38 to 4.06) | 0.002 |
| MEARS: self-discipline (standardised) | 2.98 (1.46 to 6.05) | 0.003 |
| MEARS: self-esteem (standardised) | 2.34 (1.34 to 4.10) | 0.003 |

Table S14: Results from univariable logistic regression with a ‘speeding’ coding as the outcome

| **Outcome: other motoring offence (N = 14379, n=167)** | | |
| --- | --- | --- |
| **Predictor** | **Odds ratio (95% CI)** | **p-value** |
| Male | 2.30 (1.67 to 3.15) | <0.001 |
| Over 20 years of age at UKCAT sitting | 2.22 (1.64 to 3.02) | <0.001 |
| Abstract Reasoning (standardised) | 0.80 (0.68 to 0.85) | 0.010 |
| Decision Analysis (standardised) | 0.75 (0.66 to 0.93) | 0.004 |
| Quantitative Reasoning (standardised) | 0.86 (0.73 to >1.00) | 0.062 |
| MEARS: self-discipline (standardised) | 1.80 (1.05 to 3.08) | 0.033 |
| Total UKCAT score (standardised) | 0.81 (0.69 to 0.86) | 0.012 |
| Best of three A levels | <1.00 (0.99 to >1.00) | 0.055 |

Table S15: Results from univariable logistic regression with a ‘motoring’ coding, excluding ‘driving without due care, speeding, driving without insurance or tax, and parking fines’, as the outcome

| **Outcome: physical health (N = 14379, n=189)** | | |
| --- | --- | --- |
| **Predictor** | **Odds ratio (95% CI)** | **p-value** |
| Non-selective secondary school | 1.54 (1.08 to 2.19) | 0.018 |
| UK | 2.00 (1.02 to 3.92) | 0.043 |
| Over 20 years of age at UKCAT sitting | 0.74 (0.52 to 1.04) | 0.079 |
| Best of 3 A levels | <1.00 (0.99 to >1.00) | 0.091 |

Table S16: Results from univariable logistic regression with a ‘physical health’ coding as the outcome

**C.1: Additional results from multivariable analyses: declared outcomes**

Multivariable models were constructed using backwards stepwise selection. Unless stated otherwise, no non-cognitive scales were included in the multivariable modelling process. Note that the backwards stepwise selection resulted in empty models for the outcomes ‘any FtP declaration’ and ‘caution or conviction’.

| **Outcome: conduct (N=10117, n=25)** | | |
| --- | --- | --- |
| **Predictor** | **Odds ratio (95% CI)** | **P-value** |
| Male sex | 8.45 (2.50 to 28.53) | 0.001 |
| UK resident | 0.02 (0.003 to 0.21) | 0.001 |

Table S17: Results from the backwards stepwise multivariable logistic regression model with ‘conduct’ as the outcome

| **Outcome: current or future proceedings (N=10117, n=9)** | | |
| --- | --- | --- |
| **Predictor** | **Odds ratio (95% CI)** | **P-value** |
| Male sex | 9.78 (1.08 to 71.54) | 0.042 |
| Non-professional background | 13.78 (3.28 to 57.86) | <0.001 |

Table S18: Results from the backwards stepwise multivariable logistic regression model with current or future proceedings as the outcome.

| **Outcome: disciplinary action by employer (N=7621, n=6)** | | |
| --- | --- | --- |
| **Predictor** | **Odds ratio (95% CI)** | **P-value** |
| Best of three A-level | 0.99 (0.98 to <1.00) | 0.003 |

Table S19: Results from the backwards stepwise multivariable logistic regression model with ‘disciplinary’ action by employer as the outcome, where best-of-three A-level score was included in the initial model

| **Outcome: fined or warned by regulator (N=10117, n=10)** | | |
| --- | --- | --- |
| **Predictor** | **Odds ratio (95% CI)** | **P-value** |
| UK resident | 0.006 (0.001 to 0.059) | <0.001 |

Table S20: Results from the backwards stepwise multivariable logistic regression model with ‘fined or warned by regulator’ as the outcome.

| **Outcome: fixed penalty notice (N=10117, n=213)** | | |
| --- | --- | --- |
| **Predictor** | **Odds ratio (95% CI)** | **P-value** |
| Male sex | 3.64 (2.42 to 5.47) | <0.001 |
| Age ≥ 20 at UKCAT sitting | 2.11 (1.28 to 3.48) | 0.003 |
| Non-White ethnicity | 0.63 (0.41 to 0.96) | 0.032 |

Table S21: Results from the backwards stepwise multivariable logistic regression model with ‘fixed penalty notice’ as the outcome.

| **Outcome: formal disciplinary action taken by medical school or university** | | |
| --- | --- | --- |
| **Predictor** | **Odds ratio (95% CI)** | **P-value** |
| Male sex | 3.40 (1.41 to 8.17) | 0.006 |
| Verbal Reasoning score | 0.59 (0.40 to 0.86) | 0.006 |
| EPM | 0.35 (0.15 to 0.82) | 0.016 |
| IVQ33/ITQ50: aloofness | 1.79 (1.24 to 2.58) | 0.002 |

Table S22: Results from the backwards stepwise multivariable logistic regression model with ‘formal disciplinary action taken by medical school’ as the outcome, where the ‘aloofness’ and ‘narcissism’ scales of the IVQ33/ITQ50 were included in the initial model

| **Outcome: penalty notice for disorder or harassment (N=10117, n=43)** | | |
| --- | --- | --- |
| **Predictor** | **Odds ratio (95% CI)** | **P-value** |
| Male sex | 8.08 (3.14 to 20.78) | <0.001 |
| UK resident | 0.038 (0.003 to 0.33) | 0.003 |

Table S23: Results from the backwards stepwise multivariable logistic regression model with ‘penalty notice for disorder or harassment’ as the outcome.

**C.2: Additional results from multivariable analyses: coded outcomes**

Note: the backwards stepwise selection resulted in an empty model for the outcome ‘other motoring offences’.

| **Outcome: speeding (N=10117, n=58)** | | |
| --- | --- | --- |
| **Predictor** | **Odds ratio (95% CI)** | **P-value** |
| EPM | 0.40 (0.17 to 0.92) | 0.031 |

Table S24: Results from the backwards stepwise multivariable logistic regression model with ‘speeding’ as the outcome.

| **Outcome: physical health (N=10117, n=138)** | | |
| --- | --- | --- |
| **Predictor** | **Odds ratio (95% CI)** | **P-value** |
| Non-selective secondary school | 1.76 (1.12 to 2.74) | 0.013 |

Table S25: Results from the backwards stepwise multivariable logistic regression model with ‘physical health’ as the outcome.
